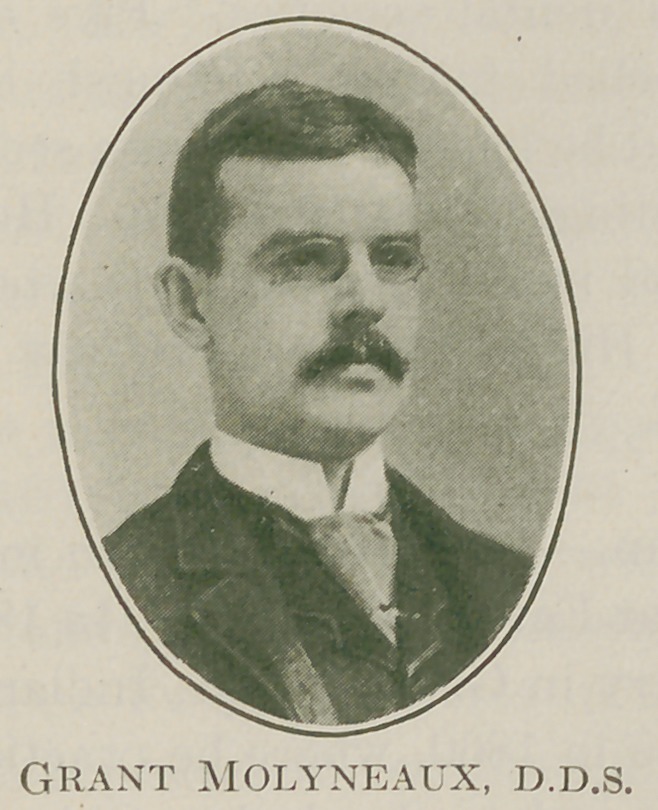# Grant Molyneaux, D.D.S.

**Published:** 1911-07-15

**Authors:** 


### OBITUARY.

GRANT MOLYNEAUX, D.D.S.

GRANT MOLYNEAUX, D.D.S. Dr. Molyneaux was born in New Richmond, Ohio, July 1st, 1863, and died in Springfield, Ohio, May 12th, 1911, of locomotor ataxia, from which he suffered for several years. He was burried at Oxford, Ohio, where his brother, Dr. John Molyneaux, practices dentistry. Dr. Molyneaux graduated from the Ohio College of Dental Surgery in 1883, and located for practice in Cincinnati, where he practiced nearly 25 years. He was a mechanical genius and became professor of Prosthetic Dentistry in the Ohio College of Dental Surgery, where he taught with much success for many years. He specialized in practice in the construction of appliances for the correction of palatal defects, and did much to make the construction of obturators practicable and within the realm of the practitioner of good mechanical skill. He took up Dr. Bonwill's anatomical method of articulating teeth with earnestness at an early date, when no one could see much in his methods, and by his genius and untiring energy and frequent papers before our state and national societies, succeeding in getting a few men in the profession to belicve in the Bonwill principles. Dr. Molyneaux was not only an earnest and indefatigable worker, but a man of fine physique, and commanding presence, with good vocabularly and a keen mind, and he enjoyed very much engaging in the general discussions in our dental societies. Five or six years ago his health compelled him to relinquish his practice and teaching work, and he has not since been seen or heard, even in the society meetings of his own state. He was a generous and kind man and made many friends who will regret his untimely death. His father, two brothers and two sisters are living.

## Figures and Tables

**Figure f1:**